# Motivating change-oriented behavior through coaching leadership: the role of psychological entitlement and knowledge management

**DOI:** 10.3389/fpsyg.2025.1626507

**Published:** 2025-09-16

**Authors:** Jiaming Hu, MyeongCheol Choi, Hann Earl Kim

**Affiliations:** Department of Business Management, Gachon University, Seongnam, Republic of Korea

**Keywords:** coaching leadership, knowledge management, psychological entitlement, change-oriented behavior, serial mediation model

## Abstract

**Introduction:**

In today’s fast-changing organizational environment, leadership styles such as coaching leadership are attracting attention for their potential to inspire innovative and proactive behaviors among employees. Coaching leadership focuses on employee development, motivation, and support, which makes it an ideal leadership style to address organizational challenges and drive change. This study examines the mediating roles of knowledge management and psychological entitlement in the relationship between coaching leadership and employee change-oriented behavior.

**Methods:**

Based on the data analysis of 492 respondents from the hospitality industry in China, this study used structural equation modeling to test the hypotheses.

**Results:**

The findings suggest that coaching leadership significantly influences knowledge management and psychological entitlement, which further mediate the role of coaching leadership on change-oriented behavior. Specifically, knowledge management provided employees with important tools and resources needed to drive change, while psychological entitlement facilitated change behavior by enhancing employees’ intrinsic motivation and responsibility.

**Discussion:**

This study deepens the understanding of the mechanisms between leadership style and change-oriented behavior, emphasizing the key roles of knowledge management and psychological entitlement. Practical implications include enhancing organizational adaptability and innovation by creating an empowering culture and optimizing systems. Future studies should expand the diversity of the sample and adopt a longitudinal research approach to capture dynamic causal relationships.

## Introduction

In today’s rapidly changing organizational environments, leadership, psychological entitlement (PE), and knowledge management (KM) are important drivers of employee behavioral transformation and organizational innovation (Ahmed and Khan, 2024; Awan and Ather, 2024). Among the many leadership styles, coaching leadership (CL) has received considerable attention in academia and practice, particularly with regard to employee development, support, and motivation (Arikan et al., 2024). It directly influences employees’ innovative and transformational behaviors by creating a supportive culture and an environment of open communication while also indirectly amplifying its impact through various mediating variables (Fang et al., 2024; Ferede et al., 2024).

Psychological entitlement and knowledge management are key mediating mechanisms connecting coaching leadership and employee change-oriented behavior (COB). Psychological entitlement is an internal psychological state that reflects employees’ perceptions of job autonomy, sense of meaning, and efficacy (Vatankhah and Raoofi, 2018). Coaching leadership can increase employees’ level of psychological entitlement by stimulating their intrinsic motivation and self-efficacy, thus enhancing their motivation and responsibility in the face of change (Rahaei and Salehzadeh, 2020).

Conversely, knowledge management, as an important practice of organizational management, provides employees with the ability and tools to drive change in complex environments by optimizing the creation and utilization of knowledge resources (Khalid et al., 2020). These two mediating variables influence employee behavior independently and work together to influence the path of coaching leadership on employee behavior.

Knowledge management is the process of creating, sharing, and applying knowledge within an organization, and its effectiveness directly impacts employees’ innovation and change behaviors (Alnaimi and Rjoub, 2019). Coaching leadership improves an organization’s overall knowledge management by creating an open culture that motivates employees to actively share and utilize knowledge (Luo and Chen, 2024). In this context, knowledge management is seen as an important bridge between coaching leadership and change-oriented behavior, indirectly facilitating employees’ willingness and ability to change by empowering them with resources and tools (Priesemuth and Taylor, 2016). Psychological entitlement, as an employee’s subjectively perceived psychological state, reflects their identification of meaning, autonomy, and competence at work (Schwarz et al., 2023). Coaching leadership significantly increases employees’ levels of psychological entitlement by clarifying goals and providing feedback and support (Moreno-Domínguez et al., 2024). When employees feel more autonomy and influence, they are more inclined to actively participate in innovation and Change-Oriented Behavior (Alnaimi and Rjoub, 2019). Psychological entitlement promotes employees’ resilience to change and increases their intrinsic drive to show higher levels of motivation and responsibility in the face of complex change situations (De Clercq and Pereira, 2023).

Although studies have been conducted to explore the mechanism of coaching leadership, knowledge management, and psychological entitlement from various aspects, most previous studies on China’s hospitality industry have focused on the developed regions of the southeast coast, paying less attention to the western region. Thus, this study, conducted in the context of the hotel service industry in Qinghai Province, has great research significance. Located in western China, Qinghai is rich in minority cultures, diverse folk traditions, and unique natural landscapes, and it has great potential for developing tourism and hotel enterprises. However, as existing research on the hotel industry in Qinghai is scarce, the special geographic and cultural environment of this region provides a new perspective and background for this study. Accordingly, based on data from the hotel industry in Qinghai, this paper reveals the deeper mechanisms by which leadership influences employee behavior by exploring the mediating roles of psychological entitlement and knowledge management between coaching leadership and change-oriented behavior.

To further strengthen the theoretical rigor of the proposed research model, this study is anchored in the Dynamic Capabilities Theory (Teece et al., 1997) and Stakeholder Theory (Freeman, 1984). The Dynamic Capabilities Theory emphasizes how organizations develop, integrate, and reconfigure internal and external competencies to address rapidly changing environments, highlighting the pivotal role of leadership and knowledge management in driving change. Stakeholder Theory, on the other hand, underscores the importance of leadership in addressing the diverse interests and needs of various organizational stakeholders through learning, adaptation, and proactive behavior. By grounding the proposed model in these theories, the inclusion and expected relationships among coaching leadership, knowledge management, and psychological entitlement are theoretically justified, enhancing the validity and generalizability of the study’s findings.

This study not only bridges the gap between regional studies but also deepens theoretical understanding and provides a reference for hotel companies in Qinghai to design more targeted management practices. The results of the study will guide leaders to optimize knowledge management practices and enhance the level of employees’ psychological entitlement, to achieve the dual enhancement of change and innovation capabilities in complex organizational environments.

## Literature review

### Coaching leadership and knowledge management

Coaching leadership, as a leadership style that focuses on employee development, plays a key role in implementing and optimizing knowledge management (Wang et al., 2021). Coaching leadership enhances employees’ self-confidence and engagement by providing the necessary resources and support, thus promoting knowledge creation and sharing (Birasnav, 2014). Especially in knowledge-intensive environments, leader behavior significantly influences employee behavior and performance in knowledge management systems (Moreno-Domínguez et al., 2024). These leaders lay the foundation for achieving organizational knowledge management goals by motivating and coaching employees in knowledge-sharing and creation activities. Moreover, coaching leadership directly contributes to the quality and willingness to use knowledge management systems (Ali et al., 2017). For example, in a study on the Iranian food industry, Jad et al. (2017) found a significant positive correlation between leaders’ knowledge-oriented behaviors and knowledge management practices. This leadership behavior not only influenced the effectiveness of knowledge management but also positively affected collaboration and knowledge-sharing behaviors among employees.

From the perspective of Dynamic Capabilities Theory, leaders play a critical role in building the organizational routines and learning processes that facilitate effective knowledge management and adaptation to change (Sampson and Swink, 2023). Coaching leadership, through its emphasis on employee development and empowerment, provides the necessary foundation for organizations to renew knowledge and respond proactively to environmental dynamics (Teece et al., 1997). Moreover, in the field of small and medium-sized enterprises (SMEs), Chaithanapat et al. (2022) emphasized that leaders help firms achieve knowledge management goals with limited resources and improve organizational innovation through knowledge sharing and utilization behaviors.

Similarly, Ali et al. (2017) noted that in healthcare settings, leaders contribute to organizational knowledge management success by promoting quality improvement of knowledge content and application of knowledge systems. Coaching leaders effectively enhance the overall level of knowledge management through explicit guidance and behavioral support. Therefore, the following hypothesis can be formulated:

*H1:* Coaching leadership has a positive effect on knowledge management.

### Coaching leadership and change-oriented behavior

As a leadership style that focuses on individual development and behavioral motivation, coaching leadership plays an important role in driving organizational change (Miao and Qian, 2016). Coaching leaders can enhance employees’ self-efficacy and behavioral autonomy by providing them with resource support and positive feedback, thus promoting change-oriented behavior (Halliwell et al., 2023). Such behaviors include innovatively proposing improved working methods, challenging existing processes, and driving organizational change efforts (López et al., 2012). At the core of change-oriented behavior lies employees’ positive adaptation and active participation in organizational change. Coaching leaders can motivate employees to be innovative and forward-thinking in complex environments through transparent communication and clear goal-setting (Kim et al., 2022). Additionally, coaching leadership strengthens employees’ acceptance and engagement in change by providing a supportive environment to help them cope with the uncertainty associated with such change (Jang, 2021). In practice, empirical research has supported the link between coaching leadership and change-oriented behavior. For example, Coaching leadership significantly enhances employees’ role breadth, self-efficacy, and constructive change responsibility, which drives employees to spontaneously engage in change-oriented behavior (Miao and Qian, 2016).

According to Stakeholder Theory, leadership behaviors that foster open communication and development address the interests of both employees and the broader organization by promoting change-oriented actions. Coaching leadership creates the trust and psychological safety needed for employees to initiate and embrace change (Freeman, 1984; Edmondson, 1999). Similarly, coaching leaders can further solidify employees’ support for change by enhancing their sense of organizational and coworker trust (Kim et al., 2022). These findings suggest that the core role of coaching leadership is to drive higher levels of behavioral performance by stimulating employees’ intrinsic motivation and sense of organizational belonging. Therefore, the following hypothesis can be formulated:

*H2:* Coaching leadership has a significant positive effect on change-oriented behavior.

### Knowledge management and psychological entitlement

As a systematic management tool, knowledge management aims to enhance the overall effectiveness of organizations and employees through the creation, sharing, storage, and application of knowledge (Nonaka and Takeuchi, 1995). Knowledge management significantly enhances employees’ sense of psychological entitlement, which is mainly expressed in the sense of trust in their ability to do their work, recognition of the meaning of their work, and recognition of their influence (Li, 2021; Soltangholi et al., 2023). In an organization where knowledge is the core resource, the effectiveness of knowledge management can directly affect psychological entitlement by enhancing employees’ self-efficacy and organizational trust (Tan et al., 2022). Psychological entitlement is a psychological state that reflects employees’ autonomy and initiative at work (Hii and Cheong, 2022). Knowledge management enhances employees’ perceived importance in the organization by motivating knowledge-sharing behaviors, which further enhances their level of psychological entitlement (Karim, 2022). Ahmed et al. (2023) suggested that knowledge sharing not only enhances employees’ sense of personal accomplishment but also improves their sense of belonging to the organization and their sense of responsibility, which has a positive impact. Additionally, in complex organizational environments, knowledge management can strengthen the effects of psychological entitlement by creating an open cultural atmosphere, reducing the possibility of knowledge concealment, and enhancing the sense of cooperation and trust among employees (Jang, 2021). Resource-based arguments and social learning theory both suggest that effective knowledge management enhances employees’ self-efficacy, autonomy, and sense of meaning, leading to higher psychological entitlement. When employees perceive access to resources and shared knowledge, they are more likely to feel empowered and entitled to contribute (Barney, 1991; Bandura, 1977). For example, in the service industry, effective knowledge management practices can significantly reduce ineffective behaviors owing to insufficient employee psychological entitlement (Alnaimi and Rjoub, 2019). In an environment facing rapid changes and high competitive pressure, knowledge management helps employees perform their work tasks more effectively by providing systematic support and resource sharing while enhancing their sense of initiative and psychological belonging (Karim, 2022). knowledge management directly enhances employees’ identification with their abilities and works through these mechanisms. Therefore, the hypothesis can be formulated as follows:

*H3:* Knowledge management has a significant positive effect on psychological entitlement.

### Psychological entitlement and change-oriented behavior

As an important psychological state, psychological entitlement is effective in enhancing employees’ self-confidence, autonomy, and work influence, thus promoting change-oriented behavior (Chughtai and Khalid, 2022). Change-oriented behavior is spontaneous behavior that goes beyond the scope of formal duties and aims to drive changes in work methods, policies, and procedures to improve organizational effectiveness (Chiaburu et al., 2011). Psychological entitlement can significantly increase employees’ motivation and initiative to engage in change activities by giving them a stronger sense of responsibility and action (Lang et al., 2022). Psychological entitlement directly contributes to change-oriented behaviors by enhancing employees’ self-efficacy and sense of responsibility for organizational change (Bernards et al., 2023). Employees who feel that their contributions to the organization are important and meaningful are more likely to take positive action to improve the status quo and solve problems (Ahmed and Khan, 2024). Building on empowerment theory, employees with greater psychological entitlement experience higher intrinsic motivation, self-confidence, and responsibility. This sense of entitlement acts as a psychological driver for proactive and change-oriented behaviors that go beyond formal job requirements (Spreitzer, 1995; Thomas and Velthouse, 1990). Additionally, Psychological entitlement can lead to higher levels of innovation and flexibility in the face of organizational change by enhancing employees’ adaptability to uncertain environments (Ahmed and Khan, 2024; Chiaburu et al., 2011). For example, in a study of public sector organizations, psychological entitlement was significantly associated with employees’ proactive behaviors during the change process. These behaviors included making innovative suggestions, adopting a proactive problem-solving approach, and driving change in complex environments (Bernards et al., 2023). Therefore, the hypothesis can be formulated as follows:

*H4:* Psychological entitlement has a significant positive effect on change-oriented behavior.

### The mediating role of knowledge management

Coaching leadership can significantly promote the implementation of knowledge management by empowering employees with guidance and resource support, and knowledge management, as the core process of knowledge creation, sharing, and application in organizations, further promotes change-oriented behavior (Mikkelsen and Olsen, 2019). Coaching leadership enhances the effectiveness of knowledge management by stimulating a culture of knowledge-sharing and collaboration, which makes employees more willing to engage in innovation and change (Jad et al., 2017). The role of knowledge management is not only in creating and storing knowledge but also in providing employees with the tools and confidence to drive change in complex environments by facilitating the effective transfer and application of knowledge (Chiaburu et al., 2022). For example, studies in the healthcare and service industries have shown that leadership improves an organization’s ability to change by influencing the quality of knowledge content and knowledge-sharing behaviors (Ali et al., 2017; Chaithanapat et al., 2022). Specifically, coaching leaders provide employees with new ways of exploring and solving problems by enhancing the effectiveness of knowledge management practices. This approach enables employees to identify opportunities for improvement and proactively drive change at work (Donate and de Sánchez Pablo, 2015). Additionally, this research has pointed to knowledge management’s ability to translate a leader’s vision and strategy into concrete action, resulting in more directed and impactful change-oriented behavior among employees (Leana-Morales and Cuevas-Vargas, 2024). Synthesizing these findings, the following hypothesis can be formulated:

*H5:* Knowledge management plays a mediating role between coaching leadership and change-oriented behavior.

### The mediating role of psychological entitlement

Coaching leadership can significantly increase employees’ psychological entitlement by providing supportive guidance and resources, and psychological entitlement as a psychological state can further contribute to change-oriented behavior (Samson and Swink, 2023). Psychological entitlement is reflected in employees’ autonomy, efficacy, and identification with the meaning of their work, and these attributes help to increase employees’ acceptance and drive for change (Bernards et al., 2023). Coaching leaders can significantly enhance employees’ sense of psychological entitlement by creating an open communication environment and a culture that encourages innovation (Mehmood et al., 2024). Psychological entitlement provides psychological support for employees to proactively drive change by increasing their sense of control over their work tasks and belonging to the organization (Shen et al., 2025). Moreover, psychological entitlement enables employees to participate more actively in the process of organizational change, as evidenced by higher levels of innovation and initiative—a behavior often seen as an important component of change-oriented behavior (Chiaburu et al., 2011). In practice, the mediating role of psychological entitlement is supported by several studies. For example, psychological entitlement bridges the gap between leaders’ motivation and employees’ change-oriented behavior by helping employees translate leaders’ support into actual change actions (Ahmed and Khan, 2024). Similarly, psychological entitlement effectively drives organizational change by enhancing employees’ sense of responsibility and innovation, making them more inclined to propose and implement new work methods and policies (Chughtai and Khalid, 2022; Lang et al., 2022). Therefore, the hypothesis can be formulated as follows:

*H6:* Psychological entitlement mediates between coaching leadership and change-oriented behavior.

**Figure 1:**
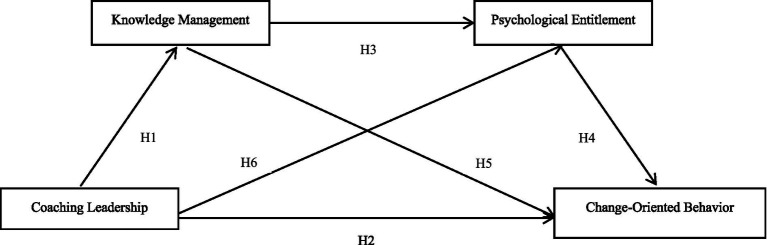
Research model.

The research hypothesis can be summarized as shown in Figure 1, which illustrates the research model.

## Methods

### Data collection

This study focuses on staff working in the hospitality industry in Qinghai Province to verify that coaching leadership significantly influences employees’ change-oriented behavior through a dual mediation path (knowledge management and psychological entitlement). It verifies the importance of leadership style on employee behavior and organizational change while highlighting the key role of knowledge management and psychological entitlement. Respondents’ positions include planning, sales, general management, research and development, production, service, technology, finance, administration, and personnel.

The study used an empirical testing methodology to analyze the survey data and explore the mediating role of psychological entitlement and knowledge management in coaching leadership and change-oriented behavior. The data were collected using an online questionnaire that spanned 2 months, from August to October 2024. With a high level of support from the hotel, employees utilized their working hours to complete the questionnaires, and 492 valid questionnaires were returned, with a valid recovery rate of 89%.

The survey results show that 52% of the respondents were male, and 38.6% were concentrated in the age range of 31–35. Regarding education, 48.4% of respondents have a bachelor’s degree, and the overall education level is high. Regarding job function distribution, 30.9% of respondents were general staff, and 39.4% were junior managers. Regarding years of work experience, 44.1% of respondents have 6–10 years of work experience. The data on company size show that 37.2% of the respondents worked in companies with 201–500 employees. Additionally, 29.9% of the parties involved were engaged in general management positions, and all parties involved were hospitality companies, with the company category being the service industry. A total of 16 hospitality companies participated in the project survey, 6 of which (37.8%) were 4-star hospitality companies, and 10 (62.2%) were 3-star hospitality companies.

The sample group has the following distinctive characteristics: it is relatively young, highly educated, with the position level dominated by general essential management, and generally has more extended working experience. These characteristics strongly support the representativeness of the research data and the reliability of the research conclusions. The unit of analysis in this study is the individual employee, as our interest centers on employee-level perceptions and behaviors within the organizational context.

### Measures

The measurement questionnaires for all variables were taken from well-established scales in authoritative international journals, and to ensure the accuracy of the meanings of each scale’s measurement entries, the English scales were translated and back-translated using Brislin’s (1970) standardized method. All questionnaires were scored on a 5-point Likert scale. In the self-assessment questionnaire, 1 stands for “strongly disagree” and 5 for “strongly agree.”

Coaching leadership is a style of leadership that focuses on supporting and guiding employees’ growth and development to achieve familiar personal and organizational goals by stimulating their potential and enhancing teamwork (Grant, 2017; Ladyshewsky, 2010). Coaching leadership centers on the leader’s ability to help employees improve their skills, increase their self-confidence, and take initiative in facing challenges through ongoing guidance and encouragement (Ellinger et al., 2003). It emphasizes building trust, open communication, and entitlement to create a positive environment that fosters personal growth and organizational innovation (Hagen, 2012; Liu and Batt, 2010). This study utilized Wang et al.’s (2021) 6-item Coaching Leadership Scale. Examples include “the level of support provided by the coaching leader in employee development” (*α* = 0.862).

Change-oriented behaviors are self-initiated behaviors that go beyond the scope of an employee’s daily duties and are designed to drive innovation and change within an organization to increase efficiency and respond to environmental changes (Choi, 2007; Wu et al., 2017). Change-oriented behaviors emphasize individuals’ initiatives to identify problems, propose solutions, and strive to improve existing work methods, processes, and systems to drive the organization to adapt to the rapidly changing external environment (Hornung and Rousseau, 2007; Zhang and Bartol, 2010). This study utilized Kim et al.’s (2022) 6-item Change-Oriented Behavior Scale. Examples include “transparency of leaders in the decision-making process” (*α* = 0.868).

Knowledge management is a systematic approach to organizational management that aims to improve employees’ and organizations’ overall effectiveness by creating, sharing, storing, and applying knowledge (Nonaka and Takeuchi, 1995). The core of knowledge management lies in making tacit knowledge explicit and transforming explicit knowledge into resources that can be widely used to promote learning, innovation, and continuous improvement within the organization (Alavi and Leidner, 2001). The 8-item knowledge management scale of Ali et al. (2017) was used in this study. Examples include “the role of leaders in promoting a culture of knowledge sharing” (α = 0.895).

Psychological entitlement is the subjective state of employees’ perceptions of competence, autonomy, and importance at work, reflecting employees’ perceptions of their influence and importance in the organization (Spreitzer, 1995). Psychological entitlement focuses on increasing employees’ sense of control and responsibility at work so that they become more confident and autonomous at work and can take the initiative to address challenges and take responsibility (Seibert et al., 2011). This study utilized the 6-item psychological entitlement scale of Lang et al. (2022). Examples include “how often employees make innovative suggestions at work” (α = 0.875).

To account for alternative explanations, we controlled for Gender, Age, and Position—three demographics frequently associated with work attitudes and behavior. Gender was included because gendered role expectations may shape leader–follower exchanges and change-related behaviors; Age and Position are linked to career stage, decision latitude, and access to information that can influence knowledge use and change actions. Measurement/coding (Table 1): Gender was coded 1 = male, 2 = female; Age was coded 1 = ≤25, 2 = 26–30, 3 = 31–35, 4 = 36–40, 5 = ≥41; Position followed ascending job levels (higher values = higher level). Thus, the mean of 1.480 for Gender corresponds to approximately 52.0% male / 48.0% female; the mean of 2.930 for Age centers the sample near the 31–35 bracket; and the mean of 2.000 for Position indicates respondents are, on average, around code 2 (low-level managers), with S.D. = 0.820 reflecting dispersion across adjacent levels. These variables were entered as covariates in the SEM and regression models without centering or transformation.

**Table 1:** Mean, standard deviations, and correlations.

Variables	Mean	S.D.	Gender	Age	Position	GL	COB	KM	PE
Gender	1.480	0.500	1						
Age	2.930	1.123	0.011	1					
Position	2.000	0.820	0.050	−0.099*	1				
CL	3.636	0.658	0.095*	−0.009	−0.039	1			
COB	3.602	0.663	0.096*	0.054	−0.011	0.654**	1		
KM	3.614	0.658	0.054	−0.042	0.064	0.615**	0.631**	1	
PE	3.640	0.676	0.075	0.041	0.008	0.675**	0.683**	0.668**	1

*N* = 492 *p < 0.05, **p < 0.01. CL, Coaching Leadership; COB, Change-Oriented Behavior; KM, Knowledge Management; PE, Psychological Entitlement.

Coding note: Gender = 1 (male), 2 (female); Age = 1 (≤25), 2 (26–30), 3 (31–35), 4 (36–40), 5 (≥41); Position = 1 (ordinary employees), 2 (low-level managers), 3 (middle-level managers), 4 (senior managers).

## Results

### Common method bias

As all focal variables were collected via self-reports at a single time point, we assessed common method variance (CMV) using Harman’s single-factor test. An unrotated EFA on all measurement items showed that the first factor explained 33.9% of the total variance—below the commonly cited 50% heuristic for problematic CMV(Podsakoff et al., 2003; Simmering et al., 2015; Chang et al., 2010). Similar results for the four construct groups (36.28, 33.32, 34.43, 34.86%) also fell below this benchmark, suggesting CMV is unlikely to be a pervasive concern in our data. We acknowledge that Harman’s test is a diagnostic, not definitive, and follow recommendations to interpret it cautiously.

### Measurement reliability and validity

Confirmatory factor analysis (CFA) was conducted on the four proposed construct models to assess the constructs’ validity and reliability. Table 2 shows the CFA results with satisfactory model fit indices (χ^2^/df = 2.401, *p* < 0.001; RMR = 0.027; GFI = 0.900; CFI = 0.939; TLI = 0.933; IFI = 0.940; RFI = 0.890; NFI = 0.901; RMSEA = 0.053). All factor loadings were highly significant (*p* < 0.001), which supports the hypothesized relationships between the constructs. Additionally, the composite reliability (CR) scores for each construct exceeded the generally accepted threshold of 0.70, indicating strong internal consistency, with coaching leadership (CR = 0.896, AVE = 0.511), change-oriented behavior (CR = 0.900, AVE = 0.523), knowledge management (CR = 0.919, AVE = 0.688), and psychological entitlement (CR = 0.906 AVE = 0.544). For each construct, the average variance extracted (AVE) values were above the recommended level of 0.50, indicating that the models had sufficient convergent validity. Moreover, discriminant validity was assessed using Fornell and Larcker’s (1981) criterion to ensure that the square root of the AVE for each construct was higher than the shared variance between it and the other constructs. The results of this analysis further confirmed the measurement model’s validity and reliability. The results of this CFA indicate that the proposed model has good measurement properties and can be used for subsequent hypothesis testing and model validation.

**Table 2:** CFA results.

Item	Estimate		S. E.	C. R.	AVE	CR	Cronbach’s α
	*β*	B					
CL6	0.724	1			0.511	0.896	0.862
CL5	0.718	0.948	0.063	15.063			
CL4	0.692	0.811	0.056	14.529			
CL3	0.727	0.972	0.064	15.248			
CL2	0.709	0.932	0.063	14.884			
CL1	0.718	0.914	0.061	15.054			
COB6	0.736	1			0.523	0.900	0.868
COB5	0.708	1.016	0.067	15.165			
COB4	0.736	1.051	0.067	15.784			
COB3	0.735	1.118	0.071	15.753			
COB2	0.705	0.97	0.064	15.099			
COB1	0.717	1.044	0.068	15.353			
KM1	0.697	1			0.688	0.919	0.895
KM2	0.693	1.153	0.081	14.294			
KM3	0.729	1.113	0.074	14.982			
KM4	0.741	1.164	0.076	15.219			
KM5	0.707	1.034	0.071	14.55			
KM6	0.736	1.192	0.079	15.12			
KM7	0.732	1.094	0.073	15.043			
KM8	0.71	1.115	0.076	14.615			
PE1	0.768	1			0.544	0.906	0.875
PE2	0.727	1.116	0.068	16.421			
PE3	0.738	1.043	0.062	16.725			
PE4	0.721	0.998	0.061	16.265			
PE5	0.739	1.018	0.061	16.73			
PE6	0.733	1.189	0.072	16.579			
Model fit: CMIN/DF = 2.401, *p* < 0.001, RMR = 0.027, GFI = 0.900, CFI = 0.939, TLI = 0.933, IFI = 0.940, RFI = 0.890, NFI = 0.901, RMSEA = 0.053							

*N* = 492 *p < 0.05, **p < 0.01. CL, Coaching Leadership; COB, Change-Oriented Behavior; KM, Knowledge Management; PE, Psychological Entitlement; C.R., critical ratio; CR, composite reliability; AVE, average variance extracted.

### Hypothesis testing

Before hypothesis testing, this study conducted a correlation analysis of the key research variables, and Table 1 shows the results. This analysis involved 492 participants and explored the relationship between demographic variables (gender, age, and position) and the key research constructs (CL, COB, KM, and PE). The study results indicated that demographic factors have limited influence on organizational behavior variables. Gender showed a significant positive correlation with coaching leadership (*r* = 0.095, *p* < 0.05) and change-oriented behavior (*r* = 0.096, *p* < 0.05), which implies that gender may have some influence on some behavioral variables. Age showed a significant positive correlation with position (*r* = 0.144, *p* < 0.01), suggesting that age has some association with career development. Between the key study variables, coaching leadership exhibited significant positive correlations with change-oriented behavior (*r* = 0.654, *p* < 0.01), knowledge management (*r* = 0.615, *p* < 0.01), and psychological entitlement (*r* = 0.675, *p* < 0.01), further emphasizing the role of coaching leadership in driving employees’ change-oriented behavior, knowledge management, and psychological entitlement. Additionally, significant positive correlations were also demonstrated between change-oriented behavior, knowledge management, and psychological entitlement (*r* = 0.683, *p* < 0.01 and *r* = 0.668, *p* < 0.01, respectively), suggesting a strong interaction between these variables. These bivariate correlations provide preliminary evidence of association only; the hypotheses are formally tested below using SEM and bootstrapped mediation analyses.

To test hypotheses 1–4, structural equation modeling (SEM) was employed in this study through AMOS 26.0. The results of the analysis are detailed in Table 3. The SEM approach provided a powerful mechanism for assessing the hypothesized associations between coaching leadership, knowledge management, psychological entitlement, and change-oriented behavior. Empirical evidence reflected through parameter estimates, standard errors, critical ratios, and significant *p*-values strongly supported all hypotheses. Good structural consistency was also indicated by the model fit indices, including CMIN/DF = 2.640, CFI = 0.929, TLI = 0.921, IFI = 0.929, RFI = 0.879, NFI = 0.890, and RMSEA = 0.058, which all indicated a satisfactory fit. Specifically, Hypothesis 1 proposed that coaching leadership positively impacts knowledge management with an estimated value of 0.638 (SE = 0.053, CR = 12.048, *p* < 0.001)—a result that emphasizes the critical role of leadership in promoting knowledge management practices. Hypothesis 2 proposed a positive effect of coaching leadership on change-oriented behavior with an estimated value of 0.374 (SE = 0.048, CR = 7.752, *p* < 0.001), which suggests that coaching leadership effectively enhances employees’ change-oriented behavior. Hypothesis 3 suggests that knowledge management has a positive effect on psychological entitlement with an estimated value of 0.854 (SE = 0.064, CR = 13.352, *p* < 0.001), indicating that knowledge management significantly enhances employees’ sense of psychological entitlement. Hypothesis 4 proposed a positive effect of psychological entitlement on change-oriented behavior with an estimated value of 0.496 (SE = 0.054, CR = 9.258, *p* < 0.001), which further supports the significant role of psychological entitlement in promoting proactive change behavior among employees. Overall, these results suggest that coaching leadership directly influences change-oriented behavior and indirectly through knowledge management and psychological entitlement. The study confirms the theoretical framework and hypotheses of the proposed model and provides solid empirical support for understanding the relationship between these constructs.

**Table 3:** The results for hypotheses 1–4.

Hypothesized path	Estimate	SE	C.R.	*p*
H1. CL → KM	0.638	0.053	12.048	***
H2. CL → COB	0.374	0.048	7.752	***
H3. KM → PE	0.854	0.064	13.352	***
H4. PE → COB	0.496	0.054	9.258	***
Model fit indices:	CMIN/DF = 2.640, *p* < 0.001, CFI = 0.929, TLI = 0.921, IFI = 0.929, RFI = 0.879, NFI = 0.890, RMSEA = 0.058			

*N* = 492 *p < 0.05, **p < 0.01, ***p < 0.001. CL, Coaching Leadership; COB, Change-Oriented Behavior; KM, Knowledge Management; PE, Psychological Entitlement; C.R., critical ratio; CR, composite reliability; AVE, average variance extracted.

**Figure 2:**
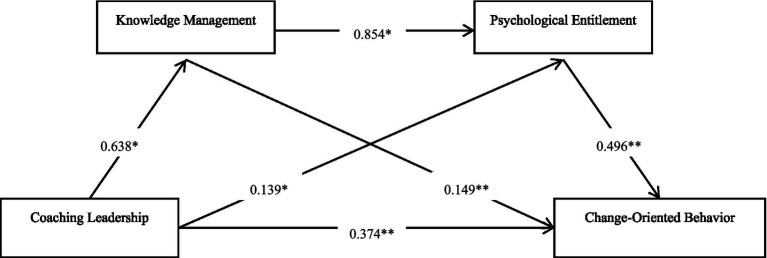
Path analysis diagram.

To verify the mediating role of knowledge management and psychological entitlement on the effect of coaching leadership on change-oriented behavior, the three-step mediation regression method of Baron and Kenny (1986) was used in this study. Table 4 shows the analysis results, which first assessed the effect of coaching leadership on knowledge management and psychological entitlement. The results of Model 1 show a significant positive correlation between coaching leadership and knowledge management (*β* = 0.658, *p* < 0.001), which fulfills the initial criterion for mediation. Similarly, Model 4 showed that coaching leadership significantly positively influenced psychological entitlement (*β* = 0.627, *p* < 0.001). This further validates the direct effect of coaching leadership on knowledge management and psychological entitlement. Next, without considering the mediating variables, Model 3 validated the direct effect of coaching leadership on change-oriented behavior (*β* = 0.589, *p* < 0.001), satisfying the necessary conditions for mediation analysis. Their significance was realized when knowledge management and psychological entitlement were used as mediating variables. Model 5 shows that knowledge management significantly affects change-oriented behavior (*β* = 0.538, *p* < 0.001), while Model 6 indicates a significant effect of psychological entitlement on change-oriented behavior (*β* = 0.411, *p* < 0.001). This suggests that coaching leadership not only significantly and positively affects change-oriented behavior, but this effect is partially realized through knowledge management and psychological entitlement. The direct effect of coaching leadership on change-oriented behavior was reduced in Model 7 (*β* = 0.327, *p* < 0.001) after introducing the mediating variables, which further emphasizes the partially mediating role of knowledge management and psychological entitlement. These results strongly support hypotheses H5 and H6, confirming the important mediating roles of knowledge management and psychological entitlement between coaching leadership and change-oriented behavior, and they provide solid empirical support for the theoretical logic of the research framework (see Figure 2) (Zhang et al., 2024).

**Table 4:** Mediating effects of KM and PE.

	H5			H6			
Variables	Model 1	Model 2	Model 3	Model 4	Model 5	Model 6	Model 7
	(KM)	(PE)	(COB)	(PE)	(KM)	(COB)	(COB)
Gender	0.86	0.097	0.134	0.103	0.070	0.134	0.120
	−0.99	−0.064	−0.045	−0.076	−0.096	−0.045	−0.037
Age	0.011	0.075	0.074	0.066	−0.005	0.074	0.068
	−0.72	0.003	−0.006	−0.013	−0.079	−0.006	−0.003
Position	0.54	0.016	0.24	0.031	0.047	0.024	0.011
	0.008	−0.024	−0.020	−0.013	0.006	−0.020	−0.003
CL	0.688***	0.510***	0.725***	0.763***	0.388***	0.725***	0.366***
	0.548	0.355	0.589	0.627	0.216	0.589	0.197
KM		0.502***					0.327***
		0.347					0.158
PE					0.538***		0.411***
					0.372		0.237
R2	0.623	0.751	0.657	0.678	0.711	0.657	0.753
Δ R2	0.388	0.564	0.432	0.459	0.506	0.432	0.567
*F* value	76.748***	125.016***	92.357***	102.96***	99.083***	92.357***	105.521***

*N* = 492; *p < 0.05, **p < 0.01, ***p < 0.001. CL, Coaching Leadership; COB, Change-Oriented Behavior; KM, Knowledge Management; PE, Psychological Entitlement.

To mediate the effect of knowledge management and psychological entitlement in the effect of coaching leadership on change-oriented behavior, the bias-corrected bootstrap method was used in this study to test the mediating effect, and the specific results are shown in Table 5. The estimated value of the total indirect effect was 0.375, and the Bootstrap 95% confidence interval was [0.286, 0.467], which significantly did not contain zero, indicating a significant indirect effect of coaching leadership on change-oriented behavior. The specific mediation pathway CL → KM → COB mediation effect estimate was 0.149 with a Bootstrap 95% confidence interval of [0.083, 0.222], accounting for 39.7% of the total indirect effect. This suggests that knowledge management played an important mediating role between coaching leadership and change-oriented behavior. The estimated CL → PE → COB mediating effect was 0.139, with a Bootstrap 95% confidence interval of [0.086, 0.202], accounting for 37.1% of the total indirect effect. The estimated mediation effect of CL → KM → PE → COB was 0.084 with a Bootstrap 95% confidence interval of [0.050, 0.126], accounting for 22.4% of the total indirect effect. This reflects that the joint mediating effect of knowledge management and PE played an important role in the effect of coaching leadership on change-oriented behavior. The estimated mediating effect of CL → PE → KM → COB was 0.077, with a Bootstrap 95% confidence interval of [0.041, 0.118], accounting for 20.5% of the total indirect effect. This pathway suggests that the combined effect of psychological entitlement and knowledge management also contributes to the conduction pathway of coaching leadership to change-oriented behavior. The results of the present study suggest that knowledge management and psychological entitlement significantly mediate the effect of coaching leadership on change-oriented behavior. Among them, the single mediating pathway (knowledge management or psychological entitlement) contributed more, while the combined mediating pathway of knowledge management and psychological entitlement also had a significant effect. These results support the hypotheses and validate the theoretical framework of the research model.

**Table 5:** The test results of mediating effects.

Hypothesis	Effect	Boot SE	Boot LLCI	BOOT ULCI	Proportion
Total indirect effect	0.375	0.046	0.286	0.467	–
CL → KM → COB	0.149	0.035	0.083	0.222	39.7%
CL → PE → COB	0.139	0.030	0.086	0.202	37.1%
CL → KM → PE → COB	0.084	0.019	0.050	0.126	22.4%
CL → PE → KM → COB	0.077	0.020	0.041	0.118	20.5%

*N* = 492; *p < 0.05, **p < 0.01. CL, Coaching Leadership; COB, Change-Oriented Behavior; KM, Knowledge Management; PE, Psychological Entitlement.

## Conclusion

### Result and discussion

This study shows that coaching leadership has a significant direct effect on employee change-oriented behavior and plays an indirect role through knowledge management and psychological entitlement. Specifically, coaching leadership significantly enhances employees’ willingness to share and apply knowledge and their views on job autonomy and meaningfulness by creating a supportive and open work environment. This further supports the critical role of leadership in driving employees to engage in innovation and change proactively.

Knowledge management plays an important mediating role between coaching leadership and change-oriented behavior. Through effective knowledge management practices, coaching leadership provides employees with the necessary resources and tools to cope with complex change situations, enhancing employees’ ability to do their jobs and increasing their willingness to change. Additionally, knowledge management accounts for 39.7% of the indirect effect in the single mediator path, which reflects its central position in driving organizational change.

Meanwhile, psychological entitlement, another key mediating variable, significantly influences change-oriented behavior by enhancing employees’ intrinsic motivation and sense of responsibility. The study reveals that employees were more inclined to propose innovations and take the initiative to drive change when they felt more meaning and autonomy in their work. The mediating path of psychological entitlement contributed 37.1% of the total indirect effect, which suggests that its role in connecting the coaching leadership with the change-oriented behavior pathway is important.

Furthermore, the joint mediation effect of knowledge management and psychological entitlement (CL → KM → PE → COB) accounted for 22.4% of the total indirect effect, reflecting the synergistic effect of the two. Knowledge management enhances employees’ competence by providing tools and resources. Psychological entitlement stimulates employees’ initiative by enhancing intrinsic drive, It plays an important role in the transmission path from coaching leadership to change-oriented behavior and provides a new perspective for the mechanism of leadership influence.

In conclusion, the study reveals the multi-level influence of coaching leadership on change-oriented behavior through direct and indirect paths, providing important insights for enterprises to optimize their management practices. By strengthening leadership training, optimizing knowledge management systems, and enhancing employees’ psychological entitlement, companies can significantly increase employees’ willingness and ability to change, thus maintaining competitiveness in a dynamic environment. By grounding the present research model in Dynamic Capabilities Theory and Stakeholder Theory, this study enhances the theoretical validity and generalizability of its findings. The results provide a robust conceptual foundation for future research on leadership, knowledge management, and change-oriented behavior across various industries and cultural contexts.

### Theoretical contribution

By integrating the mechanisms of change-oriented behavior, psychological entitlement, and knowledge management, this paper constructs a multidimensional pathway theoretical framework that enriches the research on the impact of leadership on employees’ change-oriented behaviors. This study not only reveals how coaching leadership motivates employees’ change behaviors through direct paths, but it also clarifies the indirect enhancement of employees’ propensity to change through the dual mediating roles of psychological entitlement and knowledge management, thus providing a more complex and systematic perspective for theoretical research (Bernards et al., 2023).

This study could expand the theoretical application of psychological entitlement. Although psychological entitlement has been widely studied as a motivational tool, this study clarifies its mediating effect between coaching leadership and change-oriented behavior, provides new theoretical support for the application of psychological entitlement in the field of organizational behavior, and further deepens the understanding of entitlement theory on the role of employees’ intrinsic motivation and behavior (Devi et al., 2023).

This research contributes to new perspectives in knowledge management and employee behavior research, clarifying the key mediating role of knowledge management between leadership and change-oriented behavior. This finding not only remedies the need for more attention to knowledge management influence paths in existing research but also reveals how knowledge management resource optimization enhances employees’ ability and willingness to change through leadership (Akerboom et al., 2024).

This paper fills the research gap on how multiple leadership styles collaboratively influence employee behavior. Combined with the existing literature on the separateness of leadership, this paper demonstrates how multiple leadership can promote employee behavior change through non-directive paths by introducing the bridging mechanism of knowledge management and psychological entitlement. It constructs an integrative theoretical model that provides a basis for subsequent scholars to further expand their research.

In summary, this paper’s theoretical contribution lies in its proposed integrative framework and in-depth analysis of the complex interactions between leadership, psychological entitlement, and knowledge management. This not only deepens theoretical research but also provides more comprehensive practice guidance. The integration of Dynamic Capabilities Theory and Stakeholder Theory into this study’s framework not only clarifies the mechanisms underlying the relationships among the core constructs but also supports the broader applicability of the findings. This theoretically grounded approach enables subsequent scholars to further expand and refine the research on leadership-driven change and employee behavior.

### Practical implication

This paper provides a clear guideline for management practices for companies in promoting employees’ change-oriented behavior. By verifying coaching leadership’s direct and indirect effects on employee behavior, this paper proposes a management intervention path centered on psychological entitlement and knowledge management. This finding provides theoretical support for organizations in designing incentive mechanisms and optimizing management processes in practice, which helps to enhance employees’ adaptability to and participation in organizational change and thus enhances the competitiveness of hospitality companies in dynamic environments. It reveals the central role of psychological entitlement in employee management and provides managers with specific tools to effectively motivate employees. By studying the mediating role of psychological entitlement, this paper indicates that managers can enhance employees’ innovative and proactive behaviors by increasing their sense of autonomy and trust. This provides practical suggestions for companies to build a human-oriented management culture and a basis for employees to find intrinsic motivation in their work. This research clarifies the importance of knowledge management in enhancing the organization’s overall effectiveness—especially in optimizing knowledge sharing and utilization under the guidance of leadership. The findings suggest that knowledge management enhances employees’ ability to change and supports the achievement of organizational goals through systematic knowledge flow. Therefore, enterprises can build an efficient knowledge management system to ensure the mobility and effectiveness of knowledge within the organization and take advantage of the fierce market competition.

This research provides a new direction for enterprises in leadership training and talent development. By revealing the role of coaching leadership in promoting employee behavioral change, it suggests that enterprises strengthen leadership development training for managers and exceptionally in-depth practice on how to use entitlement strategies and knowledge management tools. This will help improve managers’ leadership effectiveness and promote the entire organization’s cultural transformation. The results of this study can broaden industry applicability and improve competitiveness. Whether in manufacturing or service industries, change-oriented behavior has become one of the key competencies for organizations to cope with complex and changing environments. This research framework and recommendations provide actionable management solutions for different types of enterprises, which are of substantial practical value, especially in the face of innovation transformation or changes in the external environment.

While our recommendations are grounded in the hospitality sector in Qinghai, similar approaches can benefit other service industries and even manufacturing organizations. For instance, in a technology firm, implementing coaching leadership through regular development dialogues and peer knowledge-sharing platforms can stimulate employee initiative and adaptability. Likewise, in manufacturing, leveraging coaching leadership and knowledge management practices—such as cross-functional team coaching and best practice databases—can foster a proactive workforce ready to drive process improvements and innovation.

### Limitations and future research

Although this paper provides important contributions at the theoretical and practical levels, some limitations need to be further explored. First, this study is based on data from the hospitality industry in Qinghai Province, which has a unique regional context. China is a vast country with a total area of 9.6 million square kilometers, and the level of tourism development, cultural characteristics, and market environment in different regions show substantial differences. As a region inhabited by ethnic minorities in the West, Qinghai is rich in tourism resources, and the hospitality industry has greater development potential in the context of cultural tourism. However, directly generalizing the results of this study to developed southeast coastal regions or other tourism markets with different cultural backgrounds may have limitations. Future research could expand the sample to cover more regions and different levels of tourism markets, thus validating the applicability and generalizability of the findings.

This paper utilized a cross-sectional research design, which could not fully reveal the dynamics of causal relationships between variables. The mechanisms of leadership, knowledge management, and employee behavior in tourism management are often long-term and complex. For example, the impact of leadership may change over time and with the accumulation of employee experiences. Future research could adopt a longitudinal research approach to explore the dynamic evolution of leadership and employee behavior at different stages and the long-term impact by tracking corporate and employee data at different time points over time. Although the selection of variables in this paper focuses on the mediating roles of Psychological Entitlemen and knowledge management, it may have overlooked some factors that are particularly important in tourism management contexts, such as the organizational culture of tourism firms, service innovation capabilities, customer experience management, and the uncertainty of the external environment. These factors may have a substantial impact on the transformational behavior of employees in tourism firms. Future research could combine these key variables in the field of tourism management to construct a more systematic theoretical model that would provide a deeper understanding of the multidimensional impact of leadership on employee behavior and firm performance.

The limitations of the research methodology may affect the comprehensiveness of the results. This paper adopts a questionnaire survey and quantitative analysis methods, which provide strong empirical support for revealing the relationship between variables. However, fully explaining the deep logic behind the behavior may be difficult. Tourism management is a highly interactive and context-dependent field, and qualitative research, such as case studies and in-depth interviews, can provide more subtle insights to understand the complex mechanisms of employee behavior. Future research could adopt a mixed research methodology to shed more light on the role of coaching leadership in tourism management practices.

Overall, this study explored the mechanisms of coaching leadership, psychological entitlement, and knowledge management on employees’ change-oriented behavior in the context of the hospitality industry in Qinghai Province, providing valuable theoretical contributions and practical insights in the field of tourism management. Future research can introduce more relevant theoretical frameworks, such as paradoxical leadership theory, service-dominant logic, or service innovation theory, to further expand the understanding of tourism business management and employee behaviors to better respond to the tourism market’s ever-changing demands and challenge.

In addition to the above management recommendations, organizations are encouraged to adopt specific, evidence-based coaching techniques and structured leadership development programs to maximize the impact of coaching leadership on change-oriented behavior. For instance, leaders can implement the GROW model (Goal, Reality, Options, Will) during regular one-on-one developmental coaching sessions, helping employees clarify objectives, identify challenges, and commit to actionable change. Leadership development programs may also include workshops focused on emotional intelligence, constructive feedback, and empowerment skills—each proven to enhance employee motivation and adaptability. Furthermore, creating peer coaching circles and cross-functional mentoring programs provides ongoing learning and support, fostering a climate of collaboration and innovation. By systematically integrating these coaching tools and training initiatives into daily management practices, organizations can better equip leaders and employees to respond proactively to change, ultimately driving sustainable organizational transformation and competitiveness.

## Data Availability

The original contributions presented in the study are included in the article/supplementary material, further inquiries can be directed to the corresponding author.
