# Correction: Motivating change-oriented behavior through coaching leadership: the role of psychological entitlement and knowledge management

**DOI:** 10.3389/fpsyg.2025.1750831

**Published:** 2025-12-10

**Authors:** Jiaming Hu, MyeongCheol Choi, Hann Earl Kim

**Affiliations:** Department of Business Management, Gachon University, Seongnam, Republic of Korea

**Keywords:** coaching leadership, knowledge management, psychological entitlement, change-oriented behavior, serial mediation model

An incorrect **Funding** statement was provided. The correct funding statement reads:

“The author(s) declare that financial support was received for the research and/or publication of this article. This work was supported by the Gachon University research fund of 2023 (GCU-202303510001).”

The original version of this article has been updated.

